# Changes in *Mycobacterium tuberculosis* Genotype Families Over 20 Years in a Population-Based Study in Northern Malawi

**DOI:** 10.1371/journal.pone.0012259

**Published:** 2010-08-17

**Authors:** Judith R. Glynn, Saad Alghamdi, Kim Mallard, Ruth McNerney, Richard Ndlovu, Lumbani Munthali, Rein M. Houben, Paul E. M. Fine, Neil French, Amelia C. Crampin

**Affiliations:** 1 London School of Hygiene & Tropical Medicine, London, United Kingdom; 2 Karonga Prevention Study, Chilumba, Malawi; University of Stellenbosch, South Africa

## Abstract

**Background:**

Despite increasing interest in possible differences in virulence and transmissibility between different genotypes of *M. tuberculosis*, very little is known about how genotypes within a population change over decades, or about relationships to HIV infection.

**Methods and Principal Findings:**

In a population-based study in rural Malawi we have examined smears and cultures from tuberculosis patients over a 20-year period using spoligotyping. Isolates were grouped into spoligotype families and lineages following previously published criteria. Time trends, HIV status, drug resistance and outcome were examined by spoligotype family and lineage. In addition, transmissibility was examined among pairs of cases with known epidemiological contact by assessing the proportion of transmissions confirmed for each lineage, on the basis of IS*6110* RFLP similarity of the *M tuberculosis* strains. 760 spoligotypes were obtained from smears from 518 patients from 1986–2002, and 377 spoligotypes from cultures from 347 patients from 2005–2008. There was good consistency in patients with multiple specimens. Among 781 patients with first episode tuberculosis, the majority (76%) had Lineage 4 (“European/American”) strains; 9% had Lineage 3 (“East-African/Indian”); 8% Lineage 1 (“Indo-Oceanic”); and 2% Lineage 2 (“East-Asian”); others unclassifiable. Over time the proportion of Lineage 4 decreased from >90% to 60%, with an increase in the other 3 lineages (p<0.001). Lineage 1 strains were more common in those with HIV infection, even after adjusting for age, sex and year. There were no associations with drug resistance or outcome, and no differences by lineage in the proportion of pairs in which transmission was confirmed.

**Conclusions:**

This is the first study to describe long term trends in the four *M. tuberculosis* lineages in a population. Lineage 4 has probably been longstanding in this population, with relatively recent introductions and spread of Lineages1–3, perhaps influenced by the HIV epidemic.

## Introduction

The advent of molecular typing has provided tools for studying the relative fitness and virulence of different strains of tuberculosis [1]. Large outbreaks, and clusters of identical strains, have been taken as suggestive of virulence, and certain strains have been explored in animal or in vitro models [2], [3]. The Beijing family of strains has been most extensively investigated. While it is increasing in some areas it is stable in others, and the results from animal experiments of virulence are mixed [4], [5], [6].

Long term trends provide important evidence of relative fitness, but such data are rare [7]. Whereas an outbreak of a single strain could result from characteristics of the index patient – due to mixing patterns, cavitatory or laryngeal disease, long periods before treatment, and so on – long term trends in families of strains within populations are more likely to reflect characteristics of the strains themselves.

Most epidemiological studies have defined strains using IS*6110* RFLP, which is estimated to have a half life of about 3.5 years [8], [9]. This is ideal for contact tracing and outbreak studies, as epidemiologically related individuals are likely to share the same fingerprint. But for longer-term trends a molecular marker with a relatively slow “molecular clock” is required. Spoligotyping provides a suitable method, and families of related genotypes have already been described [10]. Each spoligotype can have a range of RFLP fingerprints, so spoligotype clusters do not necessarily represent close epidemiological linkage, and increases in a particular spoligotype in a population are unlikely to result from single outbreak events. Spoligotyping has the added advantage of being PCR-based. It can therefore be used in the absence of a live culture.

More recently different lineages of *M. tuberculosis* have been described, based on deletions and single nucleotide polymorphisms (SNPs). Some of the families described by spoligotype fit closely with these lineages, whereas Lineage 4 contains several related spoligotype families [1].

In the Karonga Prevention Study in northern Malawi we have already described RFLP based clustering results from 1996 onwards [11], [12]. Using stored sputum smears, and more recent samples, we now characterise the strains present in the population over a 20 year period from 1986, examining trends and patient characteristics over time.

## Methods

Ethics statement: The studies were approved by the Health Sciences Research Committee, Malawi and by the ethics committee of the London School of Hygiene and Tropical Medicine, UK. The specimens were collected as part of routine tuberculosis clinical activities. HIV testing was performed with counselling and consent. Written consent was sought for all later studies, including the contact study.

The Lepra Evaluation Project/Karonga Prevention Study has been carrying out population-based studies of mycobacterial disease in Karonga District, northern Malawi since the 1980s. Information on all tuberculosis cases diagnosed in the district since 1986 has been collected. Project staff are based at peripheral clinics and in the district hospital and screen individuals with chronic cough and other symptoms suggestive of tuberculosis. Diagnosed patients are interviewed, and since 1988 have been HIV tested, after counselling, and if consent is given. Procedures have been described in detail elsewhere [13]. The population of the district is now around 250,000, with 100–150 microbiologically confirmed tuberculosis cases diagnosed each year.

At least three smears are examined per patient with fluorescence microscopy, and positives confirmed on light microscopy. Since 1986 cultures have been sent to the UK for species identification and drug resistance testing. RFLP fingerprinting has been done in the UK on specimens from 1996, and spoligotyping on all cultures from late 2005 onwards [11].

Over the course of the study, smears have been stored. Many have subsequently been lost, possibly when the new laboratory was built, but 555 positive smears from the period 1986–96 remain. These were not a true random sample, but were not selected for any particular purpose so should be representative of circulating strains from the whole district.

**Figure 1:**
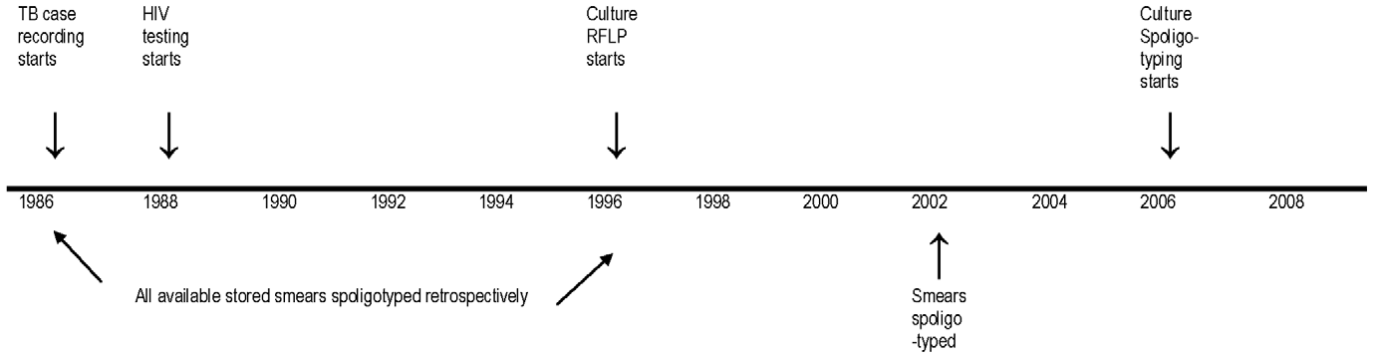
Timeline and source of isolates.

Spoligotyping [14] was performed on these archived smears using the van der Zanden protocol [15] with Chelex extraction of the DNA, and using neat, 1∶10 and 1∶100 diluted DNA. To check the reliability of results from stored smears, spoligotyping was also carried out on more recent smears from 2002. Many patients had multiple smears, and these were processed independently, blind to the patient's identity. Spoligotypes were also done on all cultures received in the laboratory from late 2005–8. Therefore spoligotype results were available from unselected patients from throughout Karonga District from 1986–2008 (figure 1).

Spoligotyping results were scanned and analysed using BioNumerics software (Applied Maths, Belgium) and checked individually by eye. Results were described by the octal code [16], and classified by comparison with the SpolDB4 database [10]. Results were also grouped into lineages based on the spoligotype [1], [17]. Analyses assessed the trends over time in lineages, spoligotype families, and spoligotypes. The characteristics of patients within each group were compared. Whether there was any evidence of variation by lineage or spoligotype was assessed overall (by chi squared test) and any associations found were assessed in more detail, using logistic regression to adjust for confounders.

As a further test of whether the lineages convey different characteristics, data on transmission in contact pairs were re-analysed. We have previously identified 143 pairs of individuals in which the first case had smear positive TB, and the second case was linked epidemiologically [18]. Epidemiological linkage was established by asking patients about contacts with previous individuals with tuberculosis and from long-standing epidemiological studies that allow close-relatives and those living in the same household to be identified. This is described in detail elsewhere [18]. We have shown that whether the second case had apparently acquired their *M. tuberculosis* from transmission from the first case – based on RFLP matching – depended on closeness of contact and HIV status of the first case. We defined transmission as confirmed if the second case in the pair had an identical RFLP pattern to the first, or if the pattern differed by 1–4 bands and the later strain was the first example of the new pattern in the population. We now categorise the strain of the first case by lineage, using data on individuals for whom both RFLP and spoligotype were available, and inferring the lineage for closely related RFLP patterns.

## Results

### Smears from 1986–1996, and 2002

Overall, spoligotype patterns were obtained from 760 smears from 518 different patients, some with multiple episodes of disease. 153 patients had multiple results from the same episode of disease, from smears taken within 3 months of each other (75 pairs, 40 triplets, 12 sets of four, one set of 5 and one of 6). Overall 128/153 (83.7%) had identical patterns among their multiple results. Only 5 patients (3.3%) had slides with totally different patterns (3 pairs, 1 triplet, 1 quadruplet) suggesting mislabelling, cross-contamination or mixed infection. The quadruplet had 2 slides showing one pattern and 2 another, suggesting mixed infection. Other patterns differed by one spacer (15 patients) 2 spacers (3), 3 spacers (1) or 4 spacers (1).

For subsequent analyses, for those with more than one smear, the more common pattern was chosen where possible. For those patients with results with similar patterns that were equally common, the pattern with more spacers was chosen, as the spoligotype patterns with different dilutions suggested that the main error was failure of amplification or hybridization to the membrane (especially for spacer 15). Four individuals were dropped from the analysis because the most likely pattern could not be inferred.

### Cultures from 2005–8

Overall 377 spoligotypes were available from 347 patients. 29 patients had multiple specimens (28 pairs, one triplet). Only one pair had completely different patterns, and was dropped. One pair had one spacer different. Two further pairs had different patterns (one had one spacer different, one completely different) but after more than 6 months.

### Multiple episodes

12 patients had results from more than one episode of TB. 6 had identical patterns (including one patient with three episodes), 3 had slight differences (one or two spacers) and 3 had completely different patterns.

### Patterns

To examine patterns and trends each patient was only included once, for their first episode of disease, and any patients with a history of previous TB were excluded. This left 781 patients.

The definition of strain families in the SpolDB4 data base is not clear: some rules are not mutually exclusive [19], and many of the patterns seen in this population have not previously been described. We have based our classification on the definitions, as shown in table 1. It is clear that some families are very similar (X and T for example), and all these related spoligotypes (LAM, H, T, X) are part of Lineage 4. Lineage 1 strains are equivalent to the spoligotype family EAI (East African Indian), Lineage 2 includes Beijing strains; Lineage 3 is equivalent to CAS [1], [17].

**Table 1:** Frequency of different Lineages and spoligotype families.

Lineage/Family	Octal code																Frequency	%
**Lineage 1 (Indo-Oceanic)**																	**61**	**7.8**
EAI	*	*	*	*	*	*	*	*	*	4	1	3	*	*	*		61	7.8
**Lineage 2 (East Asia)**																	**20**	**2.6**
Beijing	0	0	0	0	0	0	0	0	0	0	0	3	7	7	1		20	2.6
**Lineage 3 (East Africa/India)**																	**70**	**9.0**
CAS1-Kili	*	0	3	3	*	*	4	0	0	0	0	1	*	*	*		49	6.3
CAS1 other	*	0	3	*	*	*	*	4	0	0	0	3	*	*	*		21	2.7
**Lineage 4 (European-American)**																	**593**	**75.9**
H	*	*	*	*	*	*	*	*	*	*	2	0	*	*	*		11	1.4
LAM11	*	*	*	*	*	*	6	0	6	0	6	0	*	*	*		362	46.4
LAM other	*	*	*	*	*	*	6	0	7	7	6	0	*	*	*		41	5.3
T1	7	7	7	7	7	7	7	7	7	7	6	0	7	7	1		50	6.4
T other	*	*	*	*	*	*	*	7	7	7	6	0	*	*	*		60	7.7
X	*	*	*	*	*	6	7	7	7	7	6	0	*	*	*		19	2.4
Unclassified	*	*	*	*	*	*	*	*	*	*	4	0	*	*	*		50	6.4
**Lineage unclear**																	**37**	**4.7**

The definitions of spoligotype family are based on those given in SpolDB4 [21], and are shown by octal code. Only those parts of the code that define the spoligotype are shown. Other positions are shown by “*”.

EAI = East African Indian.

CAS = Central Asian.

LAM = Latin American Mediterranean.

Three quarters of the strains came from Lineage 4, with most being LAM. Overall 46% of patients had spoligotypes consistent with LAM11, of which half had spoligotype ST59 (in which all spacers are present other than those that define the LAM11 pattern). The other common spoligotypes (those present in more than 10 individuals) are shown in table 2.

**Table 2:** Trends over time for lineages, spoligotype families and the commoner spoligotypes.

Lineage Family Spoligotype			STno	1986–91		1992–96		2001–5		2006–8		Total
				n	%	n	%	n	%	n	%	
**Lineage 1 (EAI)**				8	***3.9***	4	***2.2***	12	***8.5***	37	***14.6***	61
		700777747413771	129	1	***0.5***	2	***1.1***	3	***2.1***	18	***7.1***	24
		757777777413731	806	4	***2.0***	1	***0.6***	2	***1.4***	4	***1.6***	11
**Lineage 2**												
		000000000003771	1	1	***0.5***	2	***1.1***	6	***4.3***	11	***4.3***	20
**Lineage 3 (CAS)**				8	***3.9***	8	***4.4***	16	***11.4***	38	***15.0***	70
	CAS1-Kili			2	***1.0***	2	***1.1***	11	***7.8***	34	***13.4***	49
		703377400001771	21	2	***1.0***	1	***0.6***	10	***7.1***	31	***12.2***	44
	CAS1 other			6	***2.9***	6	***3.3***	5	***3.6***	4	***1.6***	21
**Lineage 4**				175	***85.8***	159	***87.4***	106	***75.2***	153	***60.2***	593
	H			2	***1.0***	3	***1.7***	2	***1.4***	4	***1.6***	11
	LAM11			105	***51.5***	91	***50.0***	74	***52.5***	92	***36.2***	362
		777777606060771	59	32	***15.7***	24	***13.2***	35	***24.8***	60	***23.6***	151
		777777606060731	815	13	***6.4***	7	***3.9***	10	***7.1***	2	***0.8***	32
		377777606060771	59a	7	***3.4***	3	***1.7***	8	***5.7***	1	***0.4***	19
		577777606060771	59b	8	***3.9***	3	***1.7***	5	***3.6***	11	***4.3***	27
		777767606060771	59c	8	***3.9***	12	***6.6***	0	***0.0***	0	***0.0***	20
		777707606060771	59d	7	***3.4***	4	***2.2***	0	***0.0***	0	***0.0***	11
		077777606060671	1468	5	***2.5***	3	***1.7***	1	***0.7***	2	***0.8***	11
	LAM other			10	***4.9***	7	***3.9***	7	***5.0***	17	***6.7***	41
		777777607760771	42	3	***1.5***	6	***3.3***	3	***2.1***	2	***1.8***	14
		677777607760771	20	2	***1.0***	1	***0.6***	2	***1.4***	9	***3.5***	14
	T1	777777777760771	53	12	***5.9***	15	***8.2***	12	***8.5***	11	***4.3***	50
	T other			21	***10.3***	18	***9.9***	7	***5.0***	14	***5.5***	60
	X			9	***4.4***	6	***3.3***	0	***0.0***	4	***1.6***	19
	Unclassified			16	***9.1***	19	***12.0***	4	***3.8***	11	***7.2***	50
**Unclassified**				12	***5.9***	9	***5.0***	1	***0.7***	15	***5.9***	37
**Total**				204	100	182	100	141	100	254	100	781

STno. = Spoligotype numbers from the SpolDB4 database. The strains numbered here 59a–d are not found on the database.

Note that only the more common spoligotype patterns are shown for each spoligotype family. For example there were 49 individuals with CAS1-Kili, of which 44 had spoligotype 21, and 5 had other spoligotypes (not shown).

### Trends over time

Trends over time were examined by lineage, family and for the common spoligotype patterns. Time was divided into four periods to give roughly equal numbers of specimens: 1986–91, 1992–96, 2002–5, 2006–8.

**Figure 2:**
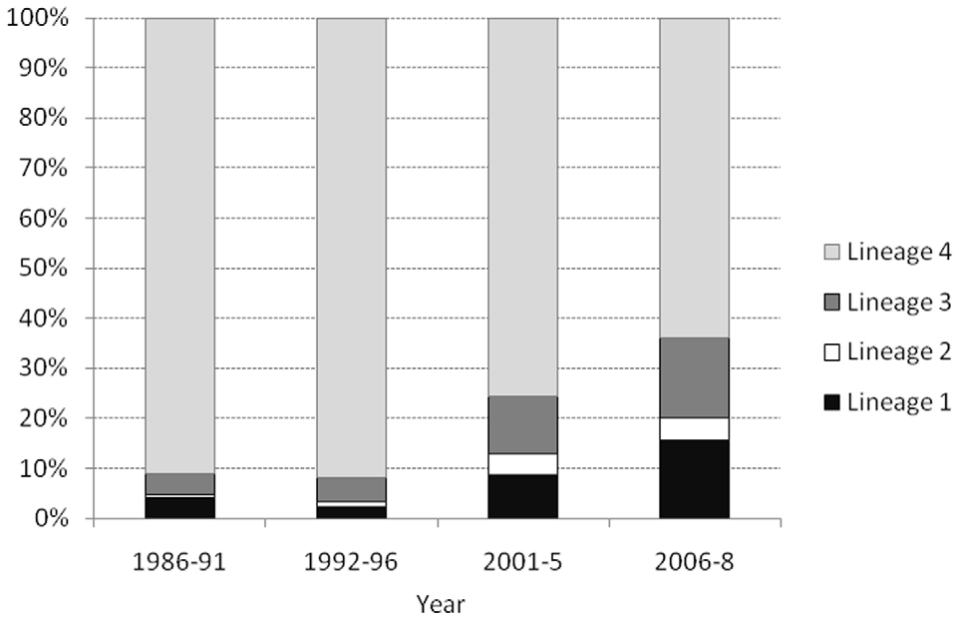
Proportion of tuberculosis due to the different lineages over time. (Isolates that could not be assigned to a lineage are excluded).

There was marked variation over time in the proportion of TB due to the different lineages (figure 2), with a decrease in Lineage 4 (from more than 90% of those with identifiable lineages in the early years, to 60% in the most recent period) and an increase in the other 3 lineages (p<0.001).

Spoligotypes in Lineage 2 other than Beijing are not clearly defined [17], so we have only considered Beijing strains. All the Beijing strains had the identical, typical spoligotype. None were found among 157 isolates from 1986–1990, with the first isolate in 1991. The proportion increased up to 4.3% (p trend = 0.003).

The increase in Lineage 3 was due to an increase in the family CAS1-Kili, and most (44/49) of the CAS1-Kili strains were a single spoligotype (ST21). The others had 3 closely related patterns. The first isolate of spoligotype ST21 was from 1987, and the proportion increased from 1% to 12% (p trend<0.001).

The Lineage 1/EAI strains were more varied: 10 different spoligotypes. The two most common patterns are shown in table 2. Spoligotype ST129 increased over time (p<0.001), and 2 closely related patterns (3 isolates) were also found in the later years. Spoligotype ST806 showed no evidence of increase, and nor did a closely related pattern found in 9 patients. Excluding spoligotype ST129, there was still some evidence of an increase over time in EAI.

Lineage 4 decreased over time. The proportion of LAM11 strains decreased in the last period, although the proportion of spoligotype ST59 increased over time. This may be an artefact, as the spoligotype that we have designated ST59c, which differs from 59 only in the absence of spacer 15 was found only up to 1994 (p<0.001), and it was previously noted that hybridisation of spacer 15 was not consistent. Another variant of the pattern, which we have called spoligotype ST59d, was not found after 1996 (p trend = 0.001).

The distribution of patients by age and sex was similar in the different lineages. None of the trends with time were changed by adjusting for age, sex or HIV status.

### Trends with HIV

HIV status was available for 615 patients: 47% were HIV positive. The proportion HIV positive varied by lineage (p = 0.007), with the highest proportion positive in Lineage 1 (68%). The association between lineage and HIV status persisted but was less strong after adjusting for year, age and sex (adjusted odds ratio for lineage 1 compared to lineage 4 = 2.10, 95% CI 1.05–4.21, table 3). The only individual spoligotype pattern that was associated with HIV status was the most common Lineage 1 spoligotype, ST129, with 17/21 (81%) HIV positive. This association was less strong, but persisted after adjusting for age, sex and year.

**Table 3:** Association of HIV status and lineage.

Lineage	HIV+ (n/N)	%	OR	OR adjusted for age, sex, year
1 (EAI)	34/50	68.0	2.76 (1.48–5.14)	2.10 (1.05–4.21)
2 (Beijing)	7/17	41.2	0.91 (0.34–2.43)	0.51 (0.18–1.43)
3 (CAS)	29/55	52.7	1.45 (0.83–2.54)	0.94 (0.50–1.74)
4	201/462	43.5	ref	ref

OR = odds ratio.

### Drug resistance

Overall 39/536 (7.3%) isolates with results available were resistant to isoniazid and 4/536 (0.75%) were resistant to rifampicin. The four patients with multidrug resistant TB were diagnosed in 1986, 1993, 1994 and 2008, and had four different spoligotype patterns. There was no evidence that the proportion of isolates that were isoniazid resistant varied by lineage, spoligotype or spoligotype family more than would be expected by chance. None of the 16 Beijing or 19 spoligotype ST129 strains tested had any drug resistance.

### Outcome

Outcome was recorded for 769 individuals: 556 were cured, 125 died, 1 had treatment failure, 55 were lost to follow-up and 32 transferred out. After excluding those who were lost, transferred or failed, the case fatality rate was 18.4%. Mortality was similar in the different lineages (8/55 (14.6%) in lineage 1, 4/17 (23.5%) in 2, 14/63 (22.2%) in 3, 93/512 (18.2%) in 4, p = 0.7). The mortality was higher in those who were HIV positive, older, had isoniazid resistance, and in earlier years. After adjusting for these factors none of the lineages or strains were significantly associated with mortality, but numbers were small (403 cases and 45 deaths with data on HIV and isoniazid resistance).

### Transmission in contact pairs

In order to define the lineage for the contact pairs we first linked the RFLP patterns to the spoligotype. Assuming that other patients in the population with the same RFLP would have the same spoligotype, we inferred the spoligotype pattern for 100 of the RFLP-defined strains from the first cases in the pairs. For the remaining cases, the likely lineage was inferred based on similarity to other RFLP-defined strains in the population. In this way the likely lineage was derived for all but 14 of the 143 patients. We used our previous definition of confirmed transmission. There was no difference by lineage of the first case in the proportion of pairs in which transmission was confirmed: 7/22 (31.8%) Lineage 1, 2/7 (28.6%) Lineage 2, 7/16 (43.8%) Lineage 3, 29/84 (34.5%) Lineage 4, (p = 0.9). There was still no difference after adjusting for closeness of contact or HIV status of the first case.

## Discussion

This small rural area of northern Malawi has examples of all the Lineages of *M. tuberculosis*. The early predominance of Lineage 4 has decreased over time, with concomitant increases in the other 3 Lineages, in particular of spoligotypes ST1 (Beijing), ST21 and ST129. The Beijing genotype is very widespread, making up the majority of tuberculosis cases in some parts of the world, and increasing in others [4]. In some countries, notably Eastern Europe, it is associated with drug resistance [4]. We have previously reported an increase in Beijing genotype in this population, based on RFLP patterns, over a shorter time period [20]. This study confirms this trend, and the lack of drug resistance in this setting, but also suggests that the proportion of tuberculosis due to the Beijing genotype has now stabilised at 4%.

Spoligotype ST21, a CAS strain, has been previously recorded (143 examples in the SpolDB4 database [21]) in Europe, the USA, eastern and southern Africa, and the Middle East. Spoligotype ST129, an EAI strain, has been recorded less frequently (19 listed in the SpolDB4 database), from Africa, Brazil, USA and Europe. Both spoligotypes ST21 and ST129 had previously been noted to be the spoligotypes of two of the common RFLP-defined clusters identified in the Karonga population [22].

The most common strain, spoligotype ST59, was the most common in each time period. The increase over time was probably an artefact due to failures of hybridisation of spacer 15 in some of the long stored samples. We have previously noted this as the most common spoligotype in our population [22], and it is common elsewhere in the region, particularly in Zambia and Zimbabwe [23], [24].

The results suggest that Lineage 4, and particularly strains with spoligotypes similar to ST59, have been well established in the area for a long time. The Beijing strains appear to be the most recent arrivals. There was evidence that Lineage 1 and 3 strains were already present in the 1980s. It is not clear if the increases are the result of chance spread following introduction or whether they represent any selective advantage. The changes may simply reflect increasing migration and travel leading to opportunities for exposure to different strains. It is intriguing that Lineage 1 strains were more commonly found in those who were HIV positive. This could suggest that the strains were less able to cause disease in those who are not immunocompromised. Lineage 1 is the predominant Lineage in South India, and it has been noted that southern Indian strains were less virulent in animal experiments [25]. In our study there were no clear associations between lineage or strain and outcome or transmission.

This is the first study to describe trends in the four *M. tuberculosis* lineages over many years in a population. We have seen clear changes in the genotype distribution, possibly in part due to the HIV epidemic.
